# Identification of GINS1 as a therapeutic target in the cancer patients infected with COVID-19: a bioinformatics and system biology approach

**DOI:** 10.1186/s41065-022-00258-5

**Published:** 2022-12-01

**Authors:** Changpeng Hu, Yue Dai, Huyue Zhou, Jing Zhang, Dandan Xie, Rufu Xu, Mengmeng Yang, Rong Zhang

**Affiliations:** Department of Pharmacy, The Second Affiliated Hospital of Army Medical University, 83 Xinqiao Road, 400037 Chongqing, China

**Keywords:** COVID-19, Cancer patients, GINS1, Bioinformatics analyses, Prognosis

## Abstract

**Background:**

Coronavirus disease 2019 (COVID-19) caused a series of biological changes in cancer patients which have rendered the original treatment ineffective and increased the difficulty of clinical treatment. However, the clinical treatment for cancer patients infected with COVID-19 is currently unavailable. Since bioinformatics is an effective method to understand undiscovered biological functions, pharmacological targets, and therapeutic mechanisms. The aim of this study was to investigate the influence of COVID-19 infection in cancer patients and to search the potential treatments.

**Methods:**

Firstly, we obtained the COVID-19-associated genes from seven databases and analyzed the cancer pathogenic genes from Gene Expression Omnibus (GEO) databases, respectively. The Cancer/COVID-19-associated genes were shown by Venn analyses. Moreover, we demonstrated the signaling pathways and biological functions of pathogenic genes in Cancer/COVID-19.

**Results:**

We identified that Go-Ichi-Ni-San complex subunit 1 (GINS1) is the potential therapeutic target in Cancer/COVID-19 by GEPIA. The high expression of GINS1 was not only promoting the development of cancers but also affecting their prognosis. Furthermore, eight potential compounds of Cancer/COVID-19 were identified from CMap and molecular docking analysis.

**Conclusion:**

We revealed the GINS1 is a potential therapeutic target in cancer patients infected with COVID-19 for the first time, as COVID-19 will be a severe and prolonged pandemic. However, the findings have not been verified actually cancer patients infected with COVID-19, and further studies are needed to demonstrate the functions of GINS1 and the clinical treatment of the compounds.

**Supplementary Information:**

The online version contains supplementary material available at 10.1186/s41065-022-00258-5.

## Introduction

The novel coronavirus disease 2019 (COVID-19) is a highly transmissible and pathogenic respiratory disease, which is caused by the severe acute respiratory syndrome coronavirus 2 (SARS-CoV-2) [1, 2]. It emerged in late 2019 and rapidly caused a global pandemic across almost all countries and territories in the world during few months [3]. In the early stage of the COVID-19 outbreak, it has been reported that individuals with underlying diseases were more susceptible to SARS-CoV-2 leading to worse outcomes [4, 5]. In recent studies, some death cases caused by COVID-19 were on account of multiple organ dysfunction syndromes rather than respiratory failure [6]. The patients with cancer, cardiovascular disease, and older age were initially identified as the riskiest factors for severe complications, even death [7–9]. What’s more, cancer patients were observed to have almost 5 times risk than patients without cancer in serious events, which accounted for a large proportion of risk factors [10, 11] .

In recent global cancer statistics, the prominent role of cancer as the second cause of death was constantly rising worldwide and constitutes an enormous burden on society [12]. The most frequently diagnosed cancers were lung, breast, colorectum, prostate, stomach, liver, esophagus, cervix, thyroid, ovary, and kidney cancer [13]. In addition, once cancer patients were infected with COVID-19, a series of genetic changes caused by COVID-19 maybe render the original treatment ineffective, which increased the difficulty of cancer clinical treatment [14, 15]. After the COVID-19 infection, the virus has been reported to cause tamoxifen resistance in breast cancer patients through blocking angiotensin-converting enzyme 2 (ACE2) [16]. It was reported that shed light on the positive correlation between ACE2 and TIL (NK cells, Dendritic cells, Neutrophils, and T-cell regulatory) which played a key role in breast cancer patients’ resistance to clinic treatment [17]. However, the study on the long-term effects of COVID-19 in cancer patients is still limited in published researches. Therefore, it is urgent to search for effective treatments for cancer patients who were infected with COVID-19.

Go-Ichi-Ni-San complex subunit 1 (GINS1, also known as PSF1) is a partner that forms a heterotetramer complex, Go-Ichi-Nii-San, with sld5, partner of sld five 2 (PSF2), and partner of sld five 3 (PSF3), respectively [18, 19]. The formation of the GINS complex is essential for eukaryotic DNA replication through recruiting Cdc45 and DNA polymerase to initiate and elongate DNA [20, 21]. GINS1 is composed of four helices, including an arch shape with a three-helical bundle (H1, H2, and H3) and an additional helix, H5. The N-terminal domain forms an arch shape, and the C-terminal domain is on top of the arch [22]. In recent studies, GINS1 was reported to involve various processes of tumor genesis and development [23, 24]. It has been demonstrated that GINS1 was highly expressed in various cancers, including intrahepatic cholangiocarcinoma, breast carcinoma, lung cancer, and colorectal cancer [24, 25]. The high expression of GINS1 was associated with several biological processes of tumor development including proliferation, invasion, and migration [26]. In addition, the depletion of GINS1 affected proliferation by blocking DNA synthesis and breaking chromosome stability in cervical cancer [27]. What’s more, the researcher found that the expression of GINS1 was significantly associated with prostate cancer grade and overall survival, which was regarded as a prognostic biomarker for prostate cancer [28]. Therefore, GINS1 was regarded as a key gene to promote tumor growth.

Interestingly, GINS1 has also been reported to be related to intracellular antigen recognition [29, 30]. Major histocompatibility complex (MHC) class I molecules was one of MHC molecules and consisted of a heavy chain (α) and a light chain (β) [31]. MHC I could present the endogenous antigen to activate cytotoxic T lymphocytes (CTL), which was significant for recognizing intracellular antigen. GINS1 (PSF1) is a transporter mediated entry of the cytosolic peptides into a pre-Golgi compartment where they bound to MHC I heavy chains [30, 32]. Then, MHC I presented bound peptides to the cell surface for antigen recognition. It has been reported that a transcriptionally inactive GINS1 gene was associated with impaired surface expression of MHC I in CTL [29]. The expression of GINS1 may be induced in the process of COVID-19 antigen recognition. GINS1 not only has the effect of anti-tumor but also can promote the antigen recognition process of cells. We hypothesis GINS1 could be a potential therapeutic target for cancer patients who are infected with COVID-19. However, whether GINS1 plays a key role in cancer patients infected with COVID-19 and the effective targeting drug remains to be further investigated.

**Fig. 1:**
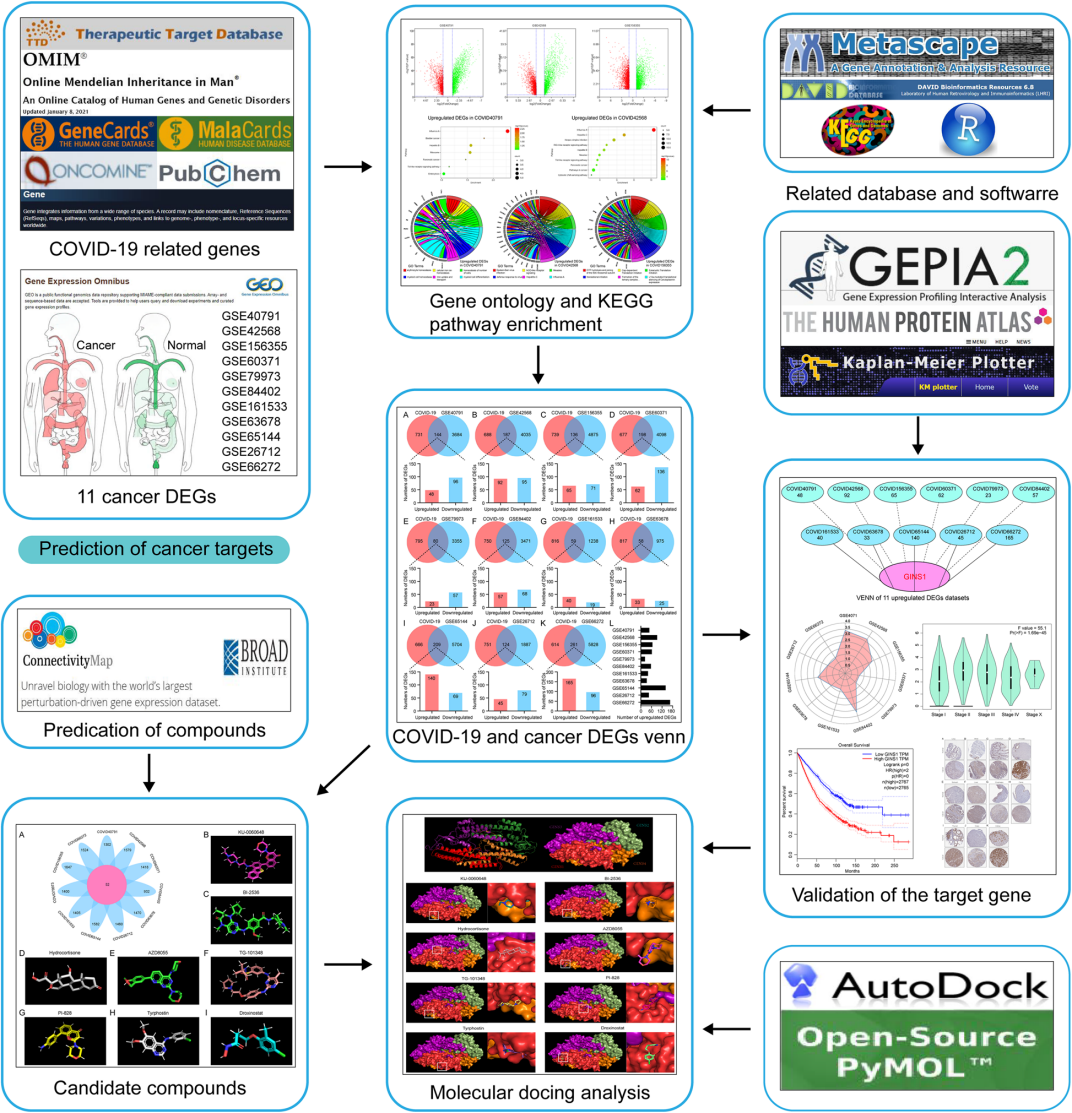
COVID-19 caused a series of biological course changes in cancer patients, which have rendered the original treatment ineffective. GINS1 was reported to promote antigen recognition and involve cancer development. GINS1 has been considered to be a promising target for the treatment of cancer patients infected with COVID-19. The eight compounds have been found to target GINS1 as potential small molecule inhibitors for the clinical treatments of cancer patients infected with COVID-19

Bioinformatics approaches are regarded as a magic weapon in biomarkers identification and drug discovery. Our previous bioinformatics analyses identified key genes that promoted the development of therapeutic resistance to erlotinib in NSCLC. Its high expression was found in both erlotinib-resistant cells and NSCLC tissues and associated with poor prognosis in patients [33]. In this study, we aim to investigate the influence of COVID-19 infection in patients with cancer by bioinformatics analyses. We firstly integrated COVID-19 related genes and identified differentially expressed genes (DEGs) of 11 types of cancer with the highest incidence. Then, we took intersections of the above genes, respectively, to acquire the targets related to COVID-19 and affected cancer patients. Additionally, Gene Ontology (GO) annotation and Kyoto Encyclopedia of Genes and Genomes (KEGG) analysis were performed to interpret the functions of these upregulated intersection genes. Simultaneously, we took a union of COVID-19 and all upregulated genes of 11 types of cancer to identify GINS1 as the key target. The high expression of GINS1 was confirmed to lead to the occurrence of tumors and affect the prognosis through the analyses of mRNA and protein expression, survival status, and expression distribution. Besides, we found 8 potential compounds in the treatment of cancer patients infected with COVID-19 by the Connectivity Map (CMap) database and molecular docking analysis. Finally, the visible graphical abstract is provided to show the flow chart of this study in cancer patients infected with COVID-19 (Fig. 1).

## Methods

### Obtaining COVID-19-associated gene set

The following databases were used to search for COVID-19-associated genes: TTD database (http://db.idrblab.net/ttd/), OMIM database (https://omim.org/), Genecards database (https://www.genecards.org/), MalaCards human disease database (https://www.malacards.org/), Oncomine database (https://www.oncomine.org/), PubChem database (https://pubchem.ncbi.nlm.nih.gov/), National Center for Biotechnology Information database (NCBI, https://www.ncbi.nlm.nih.gov/). We integrated the above search results to establish a COVID-19-associated gene set [34, 35].

### Screening of differentially expressed genes (DEGs) for cancers with the highest incidence

Eleven cancers with the highest incidence have been selected from global statistics in 2018, we obtained the datasets of these cancers in the Gene Expression Omnibus (GEO) database. The GEO accession number is GSE40791, GSE42568, GSE156355, GSE60371, GSE79973, GSE84402, GSE161533, GSE63678, GSE65144, GSE26712, and GSE66272. The differentially expressed genes (DEGs) were analyzed using an online tool GEO2R (https://www.ncbi.nlm.nih.gov/geo/geo2r/). The cut-off criteria for statistical significance were set as *p-*value < 0.01 and |log_2_FC| ≥ 1 (differential expression multiples ≥ 2), in which log_2_FC ≥ 1 was upregulated, and log_2_FC ≤ − 1 was downregulated [36].

### Gene ontology (GO) Enrichment and KEGG Analysis

Metascape (http://metascape.org/gp/index.html#/main/step1) is an online database for gene annotation and analysis. The upregulated DEGs were uploaded to this website to search for systematic information, and functional annotations of biological process (BP) were downloaded [37]. To make the enrichment of signal pathway, we subsequently conducted the Visualization and Integrated Discovery (DAVID, https://david.abcc.ncifcrf.gov/) to process the upregulated DEGs [38]. Finally, we displayed BP and KEGG signal pathway with loop plot and bubble plot.

### Expression level analysis

To confirm the expression level of GINS1 in 11 types of cancers, Gene Expression Profiling Interactive Analysis (GEPIA) (http://gepia.cancer-pku.cn/) online database was applied [39]. The lung adenocarcinoma (LUAD), lung squamous cell carcinoma (LUSC), breast invasive carcinoma (BRCA), colon adenocarcinoma (COAD), prostate adenocarcinoma (PRAD), stomach adenocarcinoma (STAD), liver hepatocellular carcinoma (LIHC), esophageal carcinoma (ESCA), cervical squamous cell carcinoma and endocervical adenocarcinoma (CESC), thyroid carcinoma (THCA), ovarian serous cystadenocarcinoma (OV), kidney Chromophobe (KICH) and kidney renal clear cell carcinoma (KIRC) datasets were selected. The cut-off criteria for statistical significance in the expression level analysis were set as *p*-value < 0.05 and |log_2_ (FC) | ≥ 2. Besides, the Human Protein Atlas (HPA, http://www.proteinatlas.org) was used to further verify the protein expression of GINS1 in normal tissues and 11 types of cancer tissues.

### Survival analysis

Moreover, the survival analysis of GINS1 in 11 types of cancers was performed using Gene Expression Profiling Interactive Analysis (GEPIA) (http://gepia.cancer-pku.cn/) [40]. The patient samples were respectively separated into a high expression group and low expression group according to GINS1 expression levels. The threshold for statistical significance in the survival analysis was set at *p*-value < 0.05 and average HR > 1.

### Prediction of candidate compounds (connectivity map)

The differentially expressed genes (DEGs) screened out from 11 databases were divided into upregulated and downregulated groups. Then, these genes were used to query the Connectivity map (https://clue.io/) database [41]. The software version is 1.1.1.43. Finally, the enrichment score representing similarity was calculated, ranging from −100 to 100. Totally 2009 compounds were related to these DEGs, and 52 compounds with a score of less than or equal to 0 were screened out from 2009 compounds. The enrichment scores of 11 databases were averaged, and the absolute value of scores which more than 50 was set as the threshold. 8 compounds were finally screened out that may be candidate drugs.

### Molecular docking of 8 candidate compounds with GINS1

Molecular docking analysis with AutoDock software (version 4.2) was utilized to further clarify binding sites and interaction forces of GINS1 protein and the 8 candidate compounds [42]. Firstly, the protein structure of GINS was downloaded from Protein Data Bank (PDB) database (https://www.rcsb.org/) and the 8 candidate compounds structure were obtained from the PubChem database (https://pubchem.ncbi.nlm.nih.gov/). Then, Open-Source PyMOL (version 2.5) software was performed for the dehydration of the receptor protein and Autodock software was used to carry out hydrogenation and charge calculation of proteins. Finally, the GINS1 molecular structure after processing was imported, and the algorithm and docking parameters were set as default. Then, we obtained and downloaded the results of molecular docking.

## Results

### Identification of COVID-19 and cancer-associated genes

Considering that the genetic changes caused by COVID-19 remain to affect cancer patients for a long time, even if their pneumonia were cured [14]. We attempted to explore the common mechanism of COVID-19 affecting the prognosis of multiple cancer patients and potential therapeutic drugs based on the genetic changes induced by COVID-19. Graphical abstract image was a flow chart illustrated the entire process of potential target identification and therapeutic drug analyses.

First, we integrated COVID-19 associated gene data sets from 7 databases (TTD, OMIM, Gene Cards, Mala Cards, Oncomine, PubChem, and NCBI gene), respectively. Deletion of duplicate genes, 875 COVID-19 associated genes were obtained. According to global cancer statistics in 2018, we selected the 11 types of cancer with the highest incidence - lung, breast, colorectum, prostate, stomach, liver, esophagus, cervix, thyroid, ovary, and kidney cancer [43]. To further explore COVID-19 susceptibility in different types of cancer patients, the expression level of infection core genes (angiotensin-converting enzyme 2 (ACE2) and transmembrane protease, serine 2 (TMPRSS2)) were evaluated in normal and tumor samples [44, 45]. The spike (S) protein of coronaviruses plays an important role in viral attacking cells [46, 47]. ACE2 as the entry receptor and TMPRSS2 as the S protein primer can promote the process of SARS-S entering the target cells [48, 49]. We proposed cancer tissues had higher mRNA expression of ACE2 and TMPRSS2 compared to normal tissues in general and may be more susceptible to SARS-CoV-2.

**Fig. 2:**
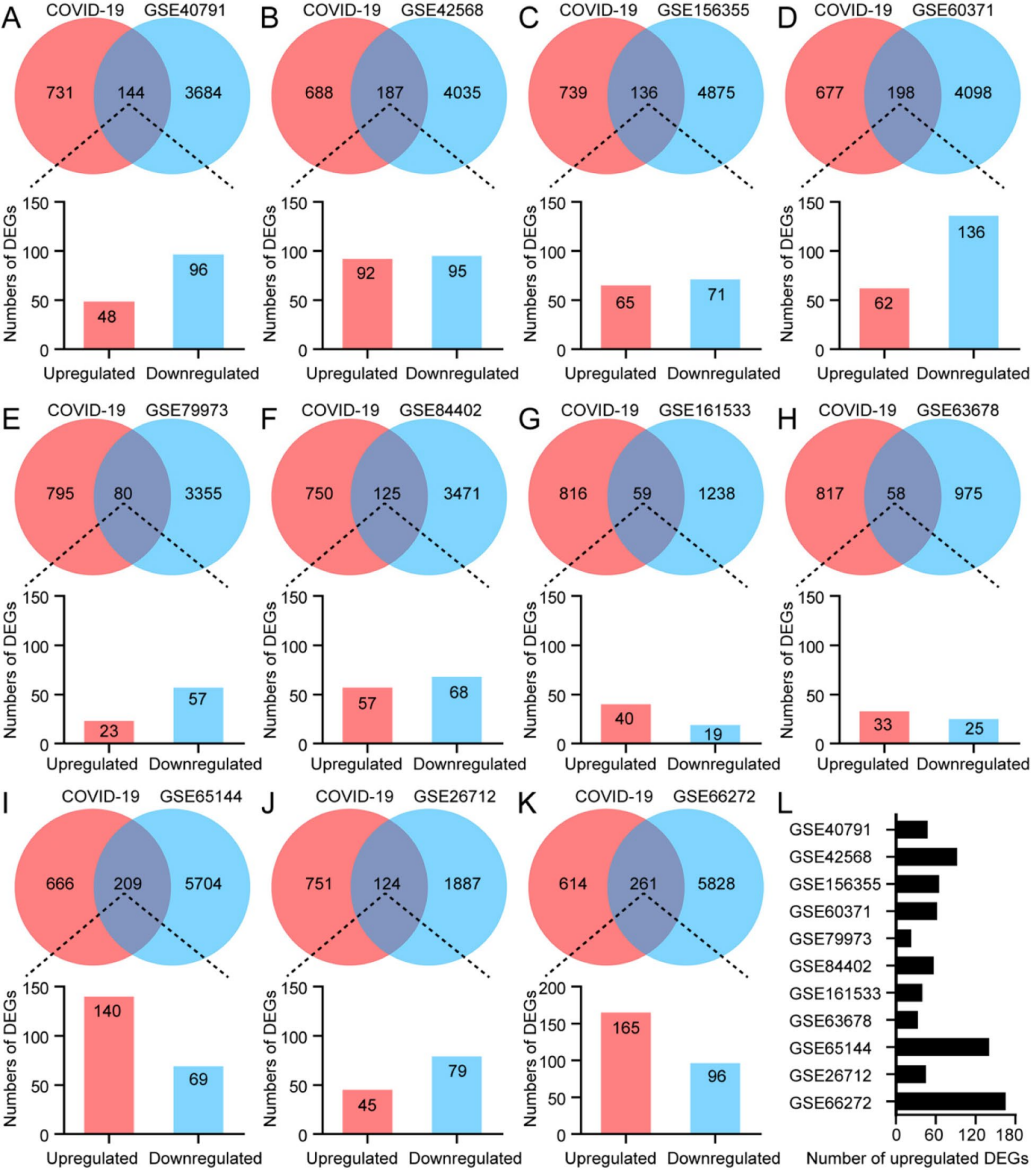
Counts of 11 types of cancer-associated DEGs. **A-K** The Venn diagram showed the intersecting targets both related to cancer patients and the patients infected with COVID-19. **L** These upregulated DEGs in each microarray were shown in a histogram. (Lung cancer: GSE40791, Breast cancer: GSE42568, Colorectal cancer: GSE156355, Prostatic cancer: GSE60371, Gastric cancer: GSE79973, Liver cancer: GSE84402, Esophagus cancer: GSE161533, Cervical cancer: GSE63678, Thyroid cancer: GSE65144, Ovarian cancer: GSE26712 and Kidney cancer: GSE66272)

Considering that cancer patients were more susceptible to COVID-19 infection, we selected 11 types of cancers with high incidence for further study. The 11 tissue microarrays datasets (GSE40791, GSE42568, GSE156355, GSE60371, GSE79973, GSE84402, GSE161533, GSE63678, GSE65144, GSE26712 and GSE66272) related to cancers screened above were downloaded from the Gene Expression Omnibus (GEO) database. GEO2R, an online analysis tool based on R language, was used to identify 11 types of cancer-associated differentially expressed genes (DEGs), respectively (Fig. S1). Furthermore, we finally acquired the key targets both related to COVID-19 and affected cancer patients by taking intersections of the COVID-19 associated genes and 11 types of cancer-associated DEGs, respectively (Fig. 2 A-K). Besides, these upregulated DEGs in each microarray were shown in Fig. 2 L. In general, we considered highly expressed genes as oncogenes that contributed to the occurrence and development of cancers [50, 51]. Taken together, bioinformatics analysis revealed that there were a large number of COVID-19-associated genetic changes in a variety of tumors. It suggested that infection with COVID-19 may lead to multiple gene changes in tumor patients. Among them, the highly expressed genes may promote tumor development, but the mechanism of these genes influencing tumor progression still needs to be further investigated.

### The biological processes and pathways involved in COVID-19 and cancer-associated genes

**Fig. 3:**
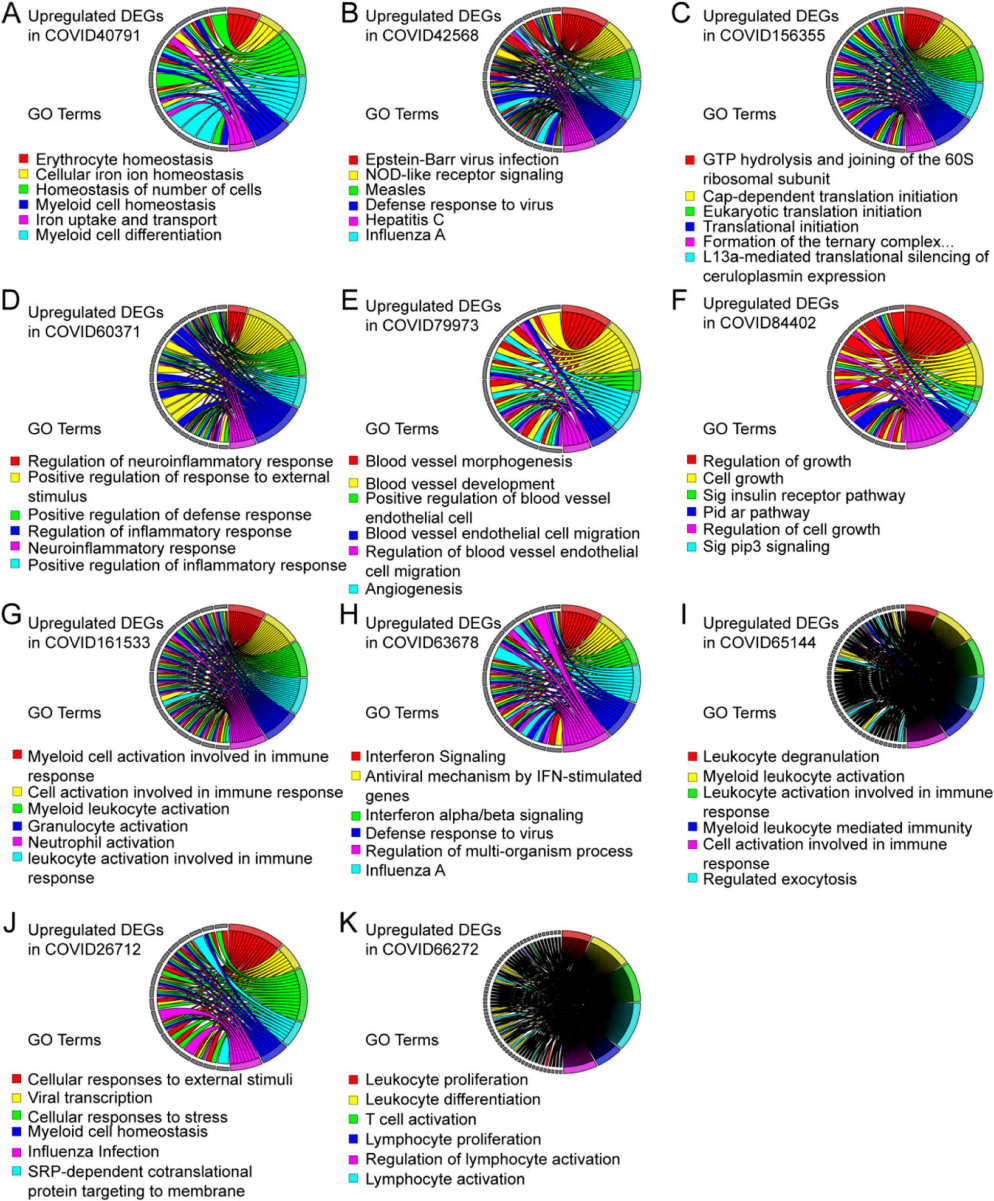
The biological processes of these upregulated intersection genes in COVID-19 and cancers were annotated by gene ontology (GO) analysis. (A: GSE40791, B: GSE42568, C: GSE156355, D: GSE60371, E: GSE79973, F: GSE84402, G: GSE161533, H: GSE63678, I: GSE65144, J: GSE26712 and K: GSE66272)

**Fig. 4:**
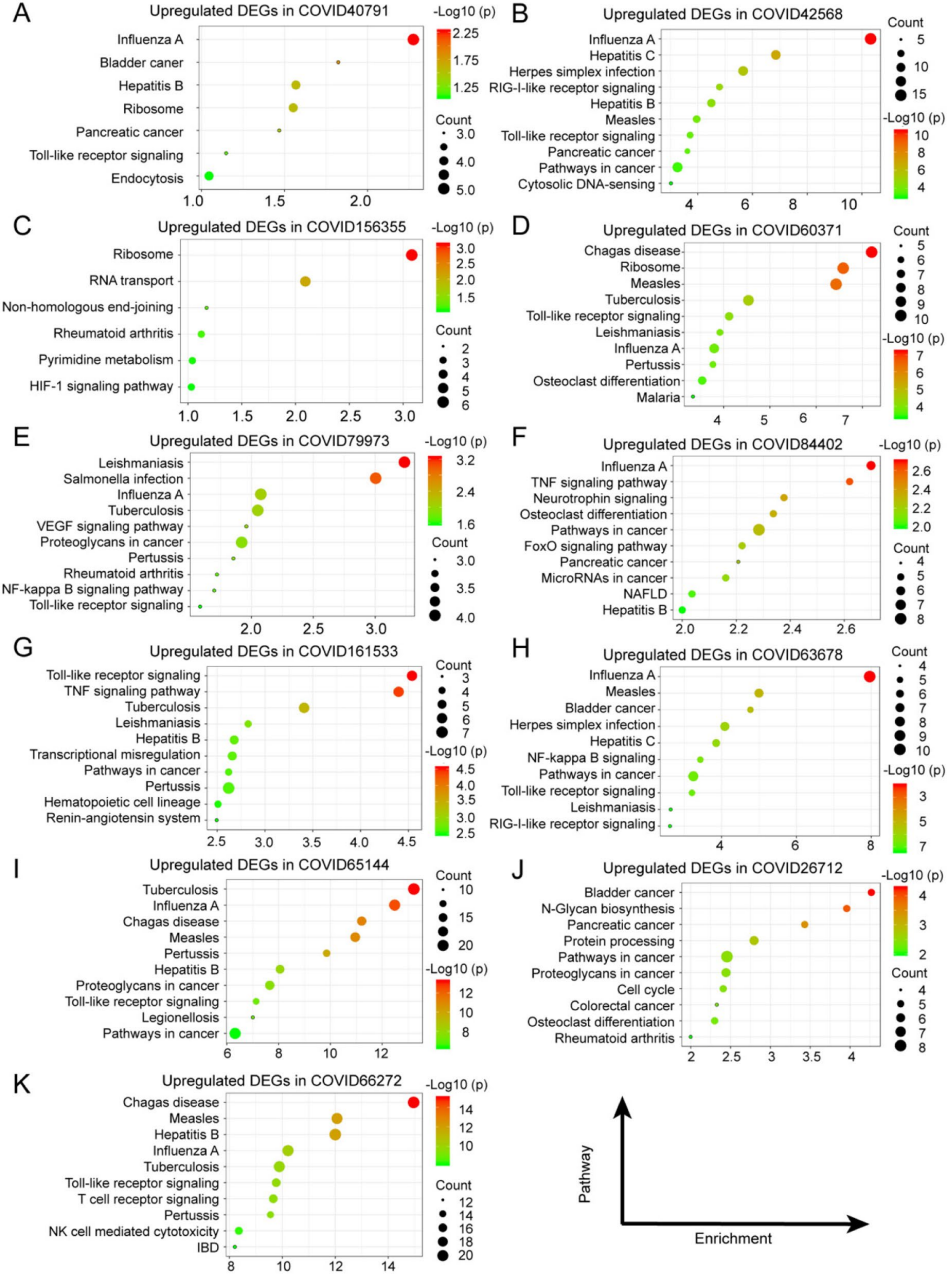
The pathway enrichments of these upregulated intersection genes in COVID-19 and cancers were annotated by Kyoto Encyclopedia of Genes and Genomes (KEGG) analysis. (A: GSE40791, B: GSE42568, C: GSE156355, D: GSE60371, E: GSE79973, F: GSE84402, G: GSE161533, H: GSE63678, I: GSE65144, J: GSE26712 and K: GSE66272)

The highly expressed genes in cancer were reported as oncogenes, which were important factors in tumor development [52, 53]. So, targeting and inhibition of these genes are significant for the clinical treatment of cancers [54, 55]. To understand the biological function involved in these upregulated intersection genes mentioned above, Gene Ontology (GO) annotation was performed respectively to discover their biological processes. As shown in Fig. 3, COVID-19 affected cancer patients mainly via regulation of virus infection and immune response, and the complete biological processes were illustrated in Supplementary Table I. Additionally, Kyoto Encyclopedia of Genes and Genomes (KEGG) analysis was performed for pathway enrichment, which indicated these upregulated intersection genes mainly involved in various virus pathways (including Influenza, tuberculosis, and pertussis) and immune-related pathways (especially in receptor signaling pathway). The 10 pathways with the highest enrichment were shown in Fig. 4 by bubble plot, and the complete pathways can be acquired in Supplementary Table II.

### Identification of the key genes of cancers infected with COVID-19

**Fig. 5:**
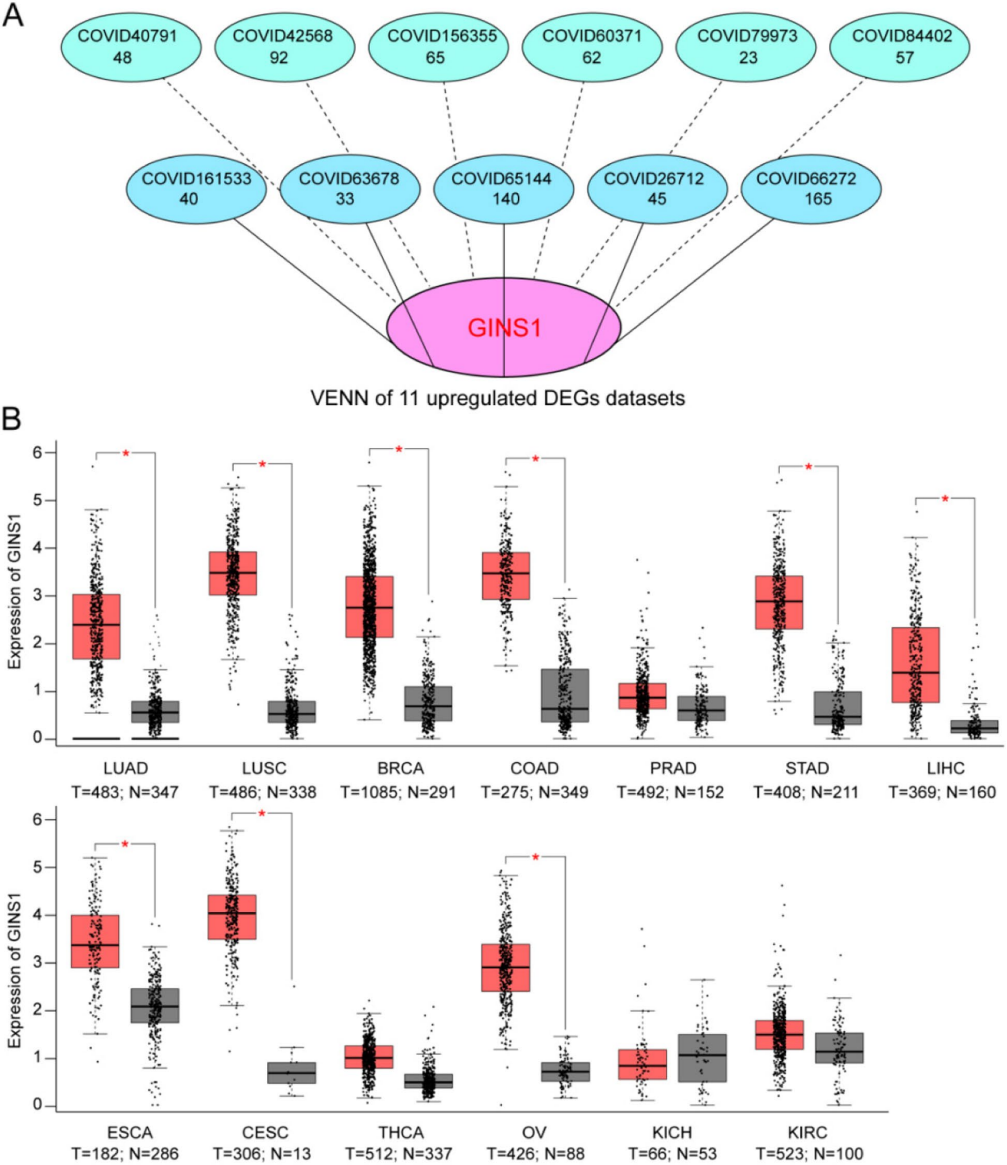
Identification of the key genes of cancers infected with COVID-19. **A** The Venn diagram of the upregulated intersection DEGs showed that GINS1 was the key target in 11 types of cancers infected with COVID-19. **B** The Box-plot represented the expression of GINS1 in 13 different types of cancers from the GEPIA database. N: normal tissues; T: tumor tissues. (LUAD: Lung adenocarcinoma, LUSC: Lung squamous cell carcinoma, BRCA: Breast invasive carcinoma, COAD: Colon adenocarcinoma, PRAD: Prostate adenocarcinoma, STAD: Stomach adenocarcinoma, LIHC: Liver hepatocellular carcinoma, ESCA: Esophageal carcinoma, CESC: Cervical squamous cell carcinoma and endocervical adenocarcinoma, THCA: Thyroid carcinoma, OV: Ovarian serous cystadenocarcinoma, KICH: Kidney Chromophobe, KIRC: Kidney renal clear cell carcinoma)

To identify the core gene targets in cancers infected with COVID-19, we took the intersection of the 11 upregulated gene sets. As shown in the Venn diagram, GINS1 was screened and identified as the key target (Fig. 5 A). It indicated that GINS1 was over-expression in cancer patients who were infected with COVID-19, and might be a potential therapeutic target for them.

### Correlation analyses of GINS1 and 11 types of cancers pathology

To understand the correlation of GINS1 and 11 types of cancers pathology, tissue expression, survival status, and expression distribution of GINS1 were analyzed. Avoiding the limitation of the low number of samples, we collected more transcription data of GINS1 in 13 different types of cancers from another platform, GEPIA, which was based on TCGA and the GTEx database contained data of 31 cancer types. As shown in Fig. 5B, a visible trend was observed that GINS1 was highly expressed in the LUAD, LUSC, BRCA, COAD, STAD, LIHC, ESCA, CESC, and OV tissue groups than in the normal tissue groups, and there was no significant difference in PRAD, THCA, KICH, and KIRC. The mRNA expression levels of GINS1 in the original 11 types of cancer datasets from GEO were displayed in Supplementary Fig. 2.

**Fig. 6:**
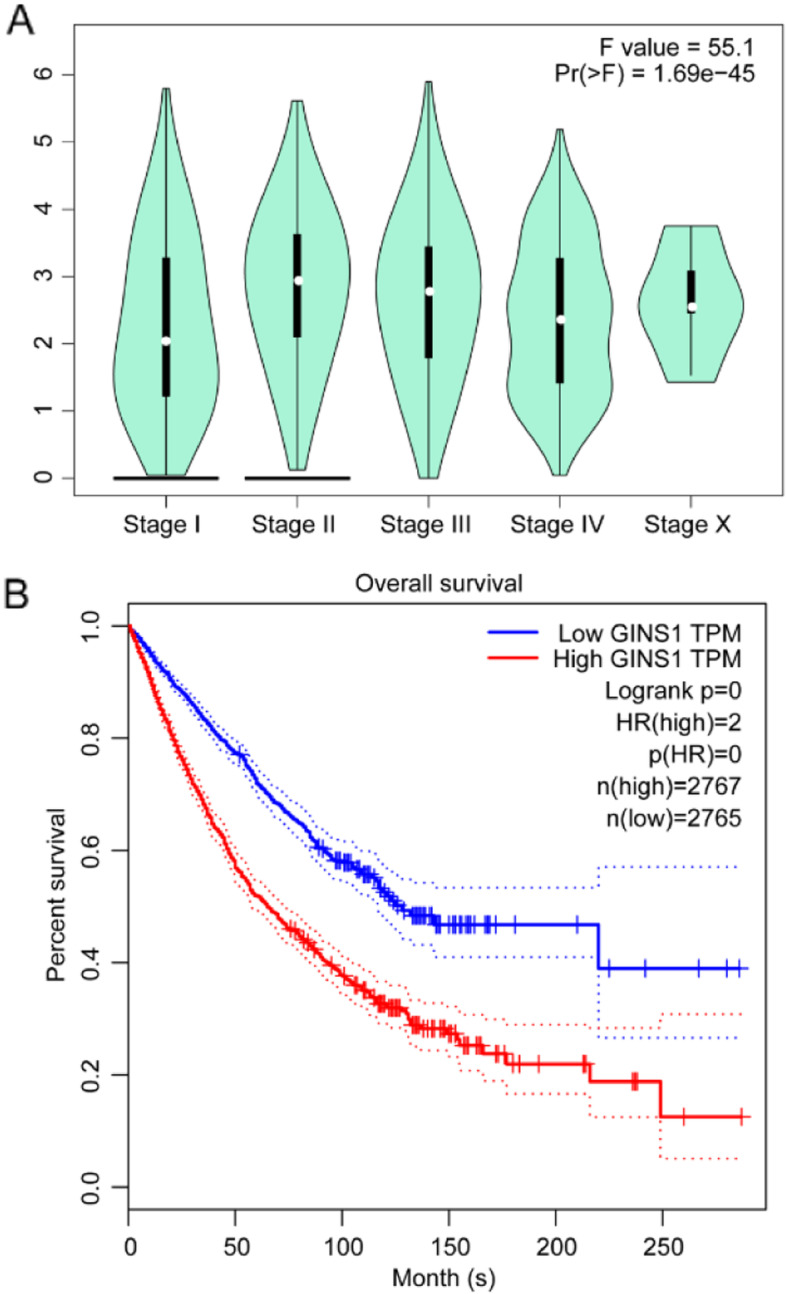
Correlation analyses of GINS1 and integral 11 types of cancers pathology. **A** The violin plot showed the correlation between GINS1 expression and the clinical staging of the 11 types of cancers in general. **B** Kaplan–Meier survival curves by GEPIA showed the correlation between GINS1 expression and overall survival of the 11 types of cancers in general

**Fig. 7:**
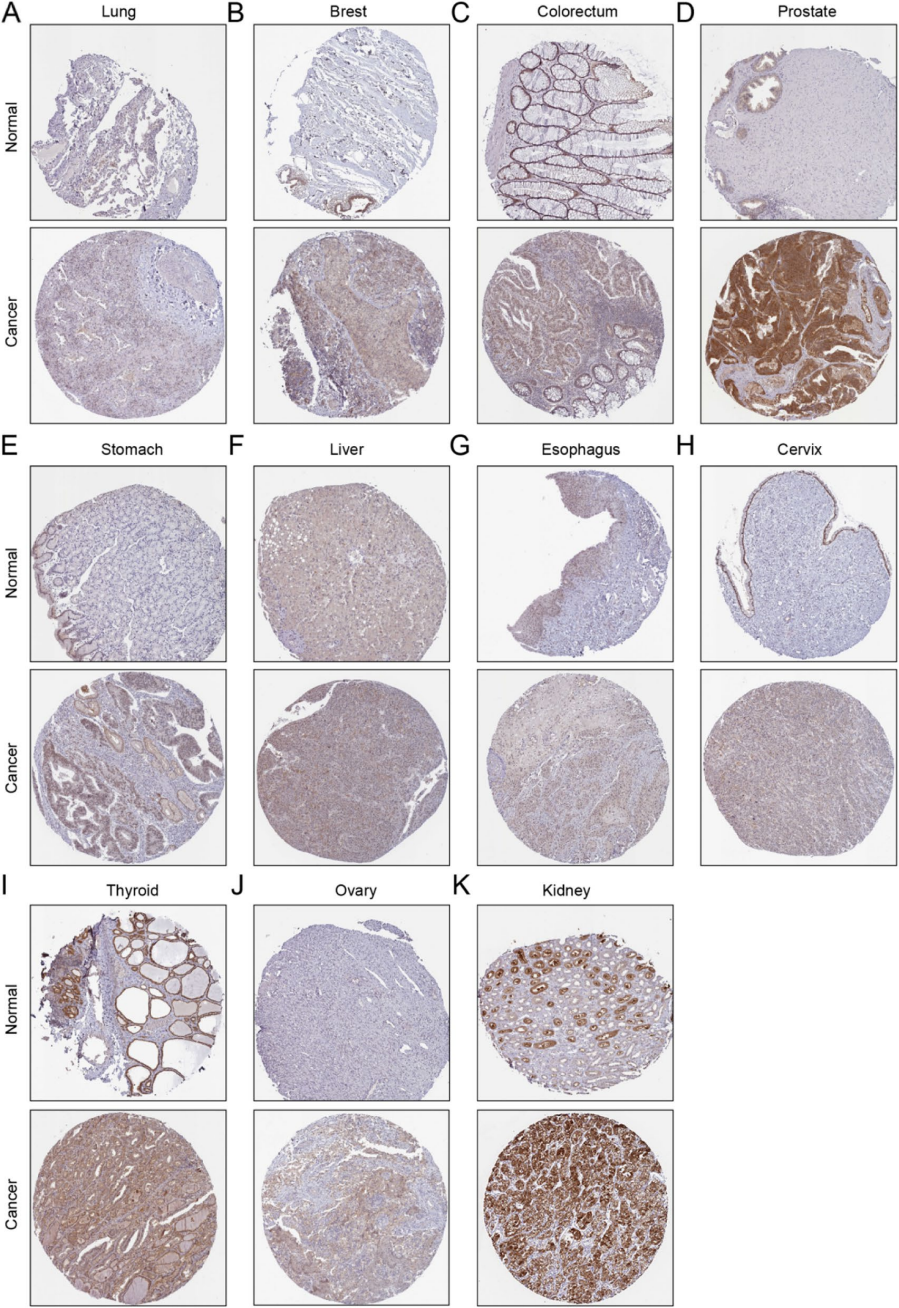
The immunohistochemistry showed the protein expression of GINS1 in 11 types of cancer and normal tissues from the Human Protein Atlas (HPA) database. (A: Lung, B: Breast, C: Colorectum, D: Prostate, E: Stomach, F: Liver, G: Esophagus, H: Cervix, I: Thyroid, J: Ovary, K: Kidney)

Next, the clinical staging analysis of the 11 types of cancers, in general, showed that the expression of GINS1 in stage I was lower compared with that in stage II, III, IV, and X. As indicated in Fig. 6 A, the expression of GINS1 was closely associated with advanced stages of cancer, may be related to cancer development or migration. Then, we investigated the GINS1 survival rate in cancers by GEPIA. The overall survival (OS) showed that the high GINS1 expression group had a low survival rate in 11 types of cancer patients in general (Fig. 6B). To further investigate the expression of GINS1 in tumors and its influence on survival time, we conducted a meta-analysis in lung cancer with the highest incidence. After the exclusion of duplicate information, 20 data sets from TCGA were considered for analyzing the expression of GINS1. After analysis, we observed that the standardized mean difference of GINS1 was higher in the cancer group than in the normal group in general (Fig. S3). Then, we investigated the GINS1 survival rate by 26 data sets. The hazard ratio showed that the GINS1 high expression group was riskier than the low expression group in general (Fig. S4). It reconfirmed that GINS1 high expressed in tumor and harm to survival time. What’s more, it implied that GINS1 expression in mRNA was a risk factor that was associated with the poor prognosis of patients. We then used the Human Protein Atlas (HPA) database to investigate the protein expression of GINS1. Notably, a significantly higher expression of GINS1 in cancer tissues was observed than in normal tissues (Fig. 7). In summary, our current results verified that GINS1 was highly expressed in cancer patients regardless of transcription or translation and risky for survival time.

### Prediction of prophylactic and protective drugs for cancer patients infected with COVID-19

**Fig. 8:**
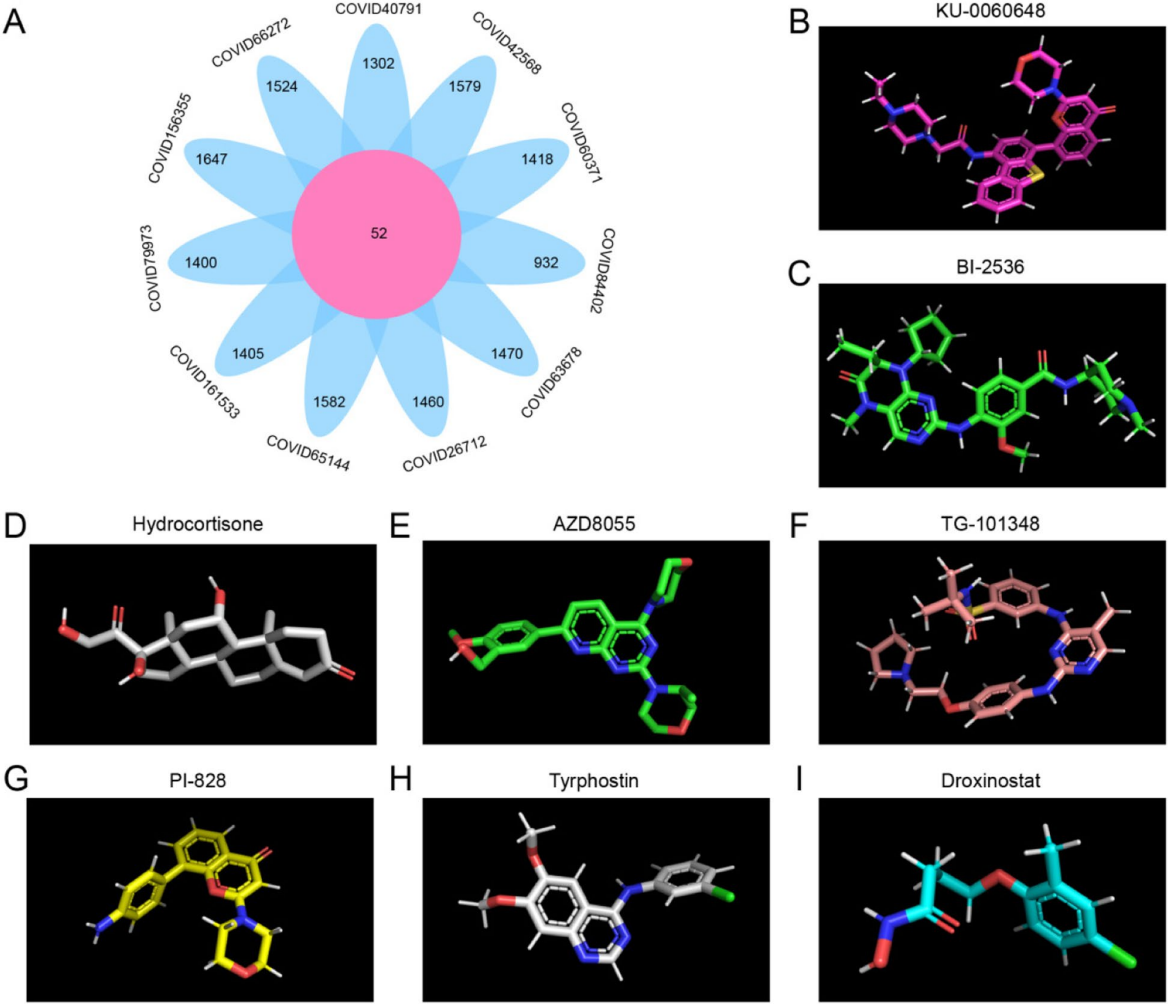
Prediction of potential compounds for cancer patients infected with COVID-19. **A** The Venn diagram showed the 52 potential compounds screened from the Connectivity Map database of DEGs in 11 types of cancer. **B-I** The molecular configuration displayed the 8 most potential compounds in the prevention and protection of cancer patients infected with COVID-19.

We have found that GINS1 expression was upregulated after COVID-19 infection in a variety of tumors. Therefore, it is significant to develop drugs targeting GINS1 for clinical treatment. We next found that there was no GINS1 specific small-molecule inhibitor reported so far. Hence our subsequent inhibitor analysis was very valuable. The Connectivity Map (CMap) is a web-based database collecting genome-wide transcriptional expression data to analyze functional connections among small molecules, gene expression changes, and pathologic processes [56]. The upregulated and downregulated DEGs in 11 types of cancer were separate to match the CMap database for searching possible agents and obtained a series of compounds. Then, we took a union of all compounds above and identified 52 compounds to be potential prevention and protection compounds in the treatment of 11 types of cancer patients infected with COVID-19 (Table SIII). The 8 most potential compounds were shown in Fig. 8.

### Prediction of binding patterns of GINS1 to 8 compounds

**Fig. 9:**
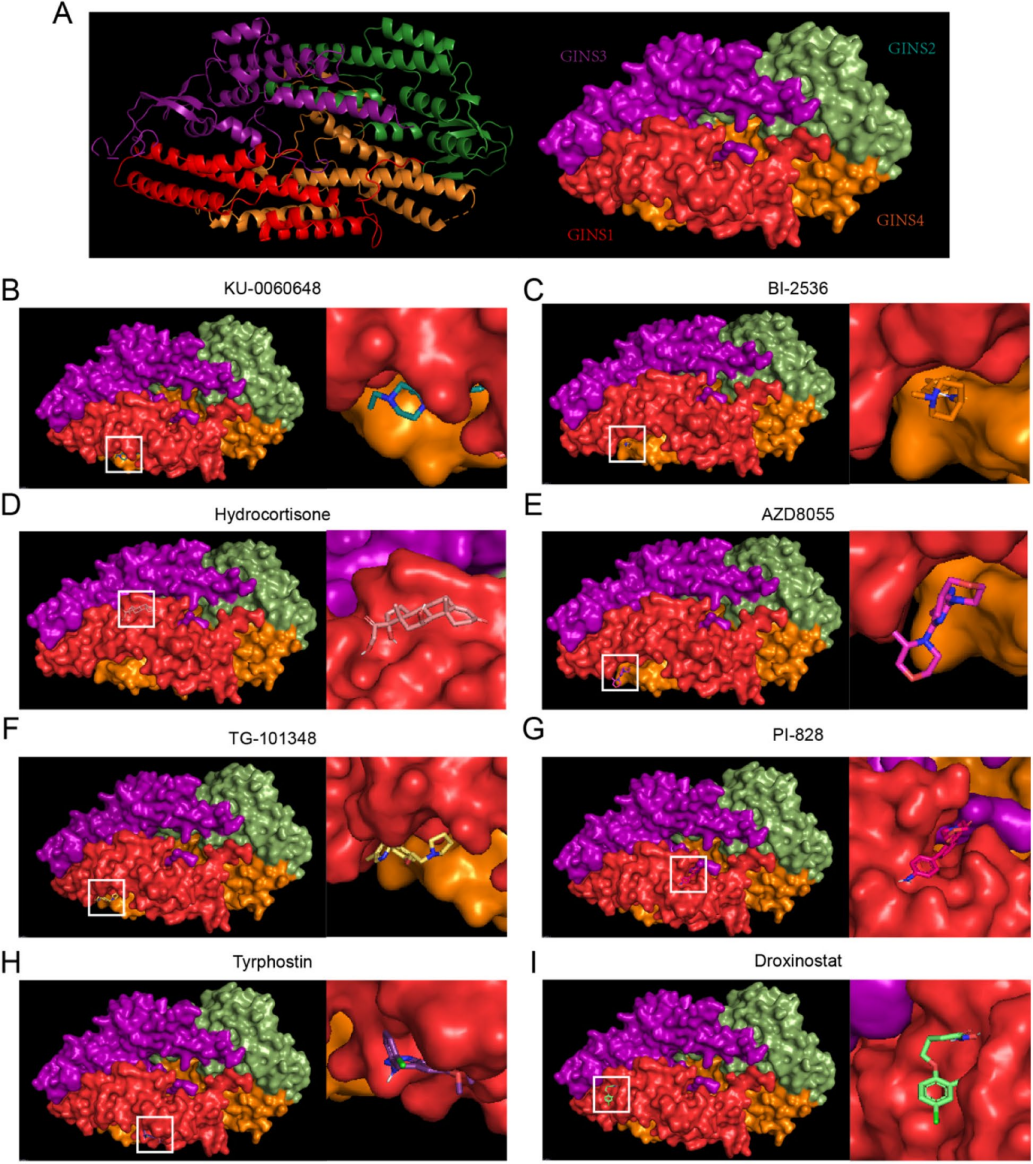
Prediction of binding patterns of GINS1 to 8 compounds. **A** The crystal structure of the GINS was collected from the PDB database. **B-I** The combinations showed that the representative binding patterns of GINS1 to 8 compounds from docking analysis, respectively

To determine the possible binding to the 8 compounds with the core target GINS1 in the cancer patients infected with COVID-19, molecular docking analysis was performed. The crystal structure of the GINS was collected from the PDB database (PDB ID 2E9X) for docking analysis with 8 compounds (Fig. 9 A). It is reported that a docking score less than −4.0 indicates a potential binding activity between the target and the compound [57]. Our results indicated that each compound can bond with the GINS1 with a high-affinity association (Table 1). One of the representative combinations with 8 compounds, respectively, was displayed in Fig. 9B-I.

**Table 1:** Molecular docking score

Molecule	Docking score(kcal/mol)
KU-0060648	-8.0
BI-2536	-6.9
hydrocortisone	-6.3
AZD8055	-6.2
TG-101348	-6.0
PI-828	-5.8
tyrphostin-AG-1478	-5.6
droxinostat	-4.8

## Discussion

COVID-19 has had and will continue to have an unprecedented impact on human health and society [58, 59]. The increasing risk of death for COVID-19 patients with cancer has raised concerns in the public [60, 61]. In recent studies, the researchers had found that when cancer patients were infected with COVID-19, 39% of cases suspended or discontinued cytotoxic chemotherapy and 5% of them changed their original chemotherapy regimen [62, 63]. Moreover, COVID-19 infection in cancer patients leads to a series of genetic changes, which may lead to original anti-tumor drug resistance and affect the prognosis of patients [14]. It has been reported that COVID-19 can cause breast cancer patients to develop tamoxifen resistance. Once breast cancer patients infected with COVID-19, the SARS-CoV-2 virus blocked ACE2, leading to impaired angiotensin production. It may induce aminopeptidase upregulation, which promotes resistance to chemotherapy in breast cancer patients [16]. Therefore, it is of great significance to explore new targets for cancer patients infected with COVID-19 and develop new targeted drugs. Our study focuses on the identification of the common mechanism of COVID-19 affecting the prognosis of cancer patients and potential therapeutic drugs based on bioinformatics analyses.

First, we have integrated the COVID-19 associated genes from seven different databases and the DEGs in each dataset of 11 different cancer types with high incidence, respectively. To establish connections of COVID-19 and different cancers, we performed biological functional annotation in the viewpoint of their shared DEGs and found virus infection and immune response played a significant role in the biological process.

To identify the key genes, we took an intersection of the upregulated crossover genes in each dataset and finally obtained GINS1. GINS1 (a partner formed the GINS complex) not only played a crucial role under physiological conditions but was also highly expressed in cancer cells and involved in cancer proliferation and migration [64]. GINS1 was required for cell proliferation in lung and colon carcinoma, and the inhibition of GINS1 suppressed the cell proliferation [64, 65]. In cell cycle regulation, GINS1-knockdown caused cell cycle arrest at the quiescent G0/G1 phase in acute myelocytic leukemia and chronic myelocytic leukemia cells [23]. During the DNA replication, the GINS complex was recruited to form a complex, CDC45-MCM-GINS (CMG), triggering the elongation of replication [21, 66]. GINS1 can affect tumor cell proliferation by regulating cell cycle checkpoints and DNA replication, so inhibiting GINS1 may be an effective strategy for tumor treatment [27, 67]. On the other hand, GINS1 has also been reported to be related to antigen recognition in T lymphocytes [29]. GINS1 could mediate the assembly of MHC I and intracellular antigen to contribute to cytotoxic T lymphocytes to recognize viral antigen [30, 68]. There have been a few reports of GINS1 promoting antigen recognition, but there was no evidence that it affects the immune system of cancer patients. Therefore, GINS1 has been considered as a promising target for the treatment of cancer patients infected with COVID-19. However, inhibitors specifically targeting GINS1 have not been found, the exploration of small molecule inhibitors is of great significance.

Considering that COVID-19 causes a large number of genetic changes in cancer patients that may affect original drug efficacy, the exploration for new therapeutic targets is urgent [14]. In this study, we revealed that the expression of GINS1 was upregulated in most tumor tissues, and correlated with tumor malignancy on the whole. At the same time, it was negatively associated with survival in overall 11 types of cancer patients. Immunohistochemistry of protein expression was further demonstrated that GINS1 was overexpressed in tumor tissues than normal tissues of 11 types of cancers, which played an important role in tumor development. Many studies used databases to analyze drugs targeting specific targets. Through CMap analysis, we identified that 52 compounds potentially have preventive and protective effects in the treatment of 11 types of cancer patients infected with COVID-19. Simultaneously, eight compounds were further identified as the most potential small-molecule inhibitors targeting GINS1. For example, hydrocortisone was been reported to support organ function in patients with severe COVID-19 [18, 69]. And tyrphostin has been identified to inhibit the replication of the influenza A virus and other viruses [70, 71]. But the effectiveness of 8 compounds in treating cancer patients infected with COVID-19 remains to be verified. We finally used molecular docking to make a preliminary prediction of the sites where these compounds might bind to GINS1. These results suggested that GINS1 may be the key target to cure cancer patients infected with COVID-19.

## Conclusion

Taken together, the bioinformatics found that GINS1 mRNA and protein levels were closely connected to patients infected with COVID-19 drawing from multiple analyses, the data was only from public databases rather than own original experiments. Therefore, the connection between GINS1 expression and COVID-19 infection in cancers needs experimental evidence. Besides, whether the 8 potential compounds could treat cancer patients infected with COVID-19 needs to be verified in further clinical trials. In this study, we provided evidence that it has a high sensitivity of COVID-19 in all the 11 types of cancers (including lung, breast, colorectum, prostate, stomach, liver, esophagus, cervix, thyroid, ovary, and kidney cancer), and GINS1 played a vital role in this process. Moreover, the expression of GINS1 in mostly cancer tissues was higher than normal tissues, providing a rationale theoretical explanation why cancer patients infected with COVID-19 could cause the more serious condition. Since COVID-19 virus infects the lung first, coupled with the high expression of GINS1 in LUAD and LUSC, we hypothesize that the cell lines of lung cancer have the highest sensitivity among these 11 cancers. In addition, we predicted potential therapeutic compounds targeting GINS1 and analyzed their binding efficiency. Our results could guide efforts for understanding the molecular mechanisms of COVID-19 infection in cancer patients. Furthermore, our study provided candidate targets and compounds on GINS1 for the clinical treatment of cancer patients infected by COVID-19.

## Supplementary information

Additional file 1. Supplementary information

## Data Availability

The RNA-sequencing datasets associated with cancers in this study were available in the public database GEO (https://www.ncbi.nlm.nih.gov/geo). The gene datasets associated with COVID-19 were available in TTD, OMIM, Gene Cards, Mala Cards, Oncomine, PubChem, and NCBI gene databases.

## Abbreviations

ACE2: Angiotensin-converting enzyme 2
BRCA: Breast invasive carcinoma
BP: Biological process
CMap: Connectivity Map
COAD: Colon adenocarcinoma
COVID-19: Coronavirus disease 2019
CTL: Cytotoxic T lymphocytes
CESC: Cervical squamous cell carcinoma and endocervical adenocarcinoma
DEGs: Differentially expressed genes
ESCA: Esophageal carcinoma
GO: Gene Ontology
GEO: Gene Expression Omnibus
GINS1: Go-Ichi-Ni-San complex subunit 1
GEPIA: Gene Expression Profiling Interactive Analysis
HPA: Human Protein Atlas
LUAD: Lung adenocarcinoma
LUSC: Lung squamous cell carcinoma
LIHC: Liver hepatocellular carcinoma
MHC: Major histocompatibility complex
OV: Ovarian serous cystadenocarcinoma
PRAD: Prostate adenocarcinoma
PSF2: Partner of sld five 2
PSF3: Partner of sld five 3
SARS-CoV-2: Severe acute respiratory syndrome coronavirus 2
STAD: Stomach adenocarcinoma
THCA: Thyroid carcinoma
TMPRSS2: Transmembrane protease serine 2
KEGG: Kyoto Encyclopedia of Genes and Genomes
KICH: Kidney Chromophobe
KIRC: Kidney renal clear cell carcinoma
