# Supplementary material for: Identification of GINS1 as a therapeutic target in the cancer patients infected with COVID-19: a bioinformatics and system biology approach
Source: Hereditas. 2022 Dec 1;159:45. doi: 10.1186/s41065-022-00258-5 (PMC9713126; doi:10.1186/s41065-022-00258-5)
Supplement: Supplementary file 1 — Supplementary information [file 41065_2022_258_MOESM1_ESM.docx]

**Supporting Information:**

**Identification of GINS1 as a therapeutic target in the cancer patients infected with COVID-19: A bioinformatics and system biology approach**

Changpeng Hu^#^, Yue Dai ^#^, Huyue Zhou, Jing Zhang, Dandan Xie, Rufu Xu, Guobing Li* and Rong Zhang*

^#^These authors contributed equally

**Authors^,^ affiliations:**

Department of Pharmacy, The Second Affiliated Hospital of Army Medical University, Chongqing 400037, China

***Corresponding authors:**

**Rong Zhang**, Department of Pharmacy, The Second Affiliated Hospital of Army Medical University, 83 Xinqiao Road, Chongqing 400037, China, Phone: 86-023-68774770 (office), E-mail: [xqpharmacylab@126.com](mailto:xqpharmacylab@126.com)

**Guobing Li,** Department of Pharmacy, The Second Affiliated Hospital of Army Medical University, 83 Xinqiao Road, Chongqing 400037, China, Phone: 86-023-68774770 (office), E-mail: [rgwlsb@126.com](mailto:rgwlsb@126.com)

**Table of content**

1. The volcano represented 11 types of cancer-associated DEGs identified by GEO2R. Red: upregulated genes; black: downregulated gene.
2. The radar map represented mRNA expression levels of GINS1 in the original 11 types of cancers from the GEO database.
3. The expression analysis of GINS1 in lung cancer was conducted by the Lung Cancer Explorer Platform.
4. The survival analysis of GINS1 in lung cancer was conducted by the Lung Cancer Explorer Platform.
5. The complete biological processes of these upregulated intersection genes in COVID-19 and cancers were annotated by GO analysis.
6. The Complete pathway enrichments of these upregulated intersection genes in COVID-19 and cancers were annotated by Kyoto Encyclopedia of Genes and Genomes (KEGG) analysis.
7. The potential prevention and protection compounds for cancer patients infected with COVID-19

**Figure S1.** The volcano represented 11 types of cancer-associated DEGs identified by GEO2R. Red: upregulated genes; green: downregulated gene.


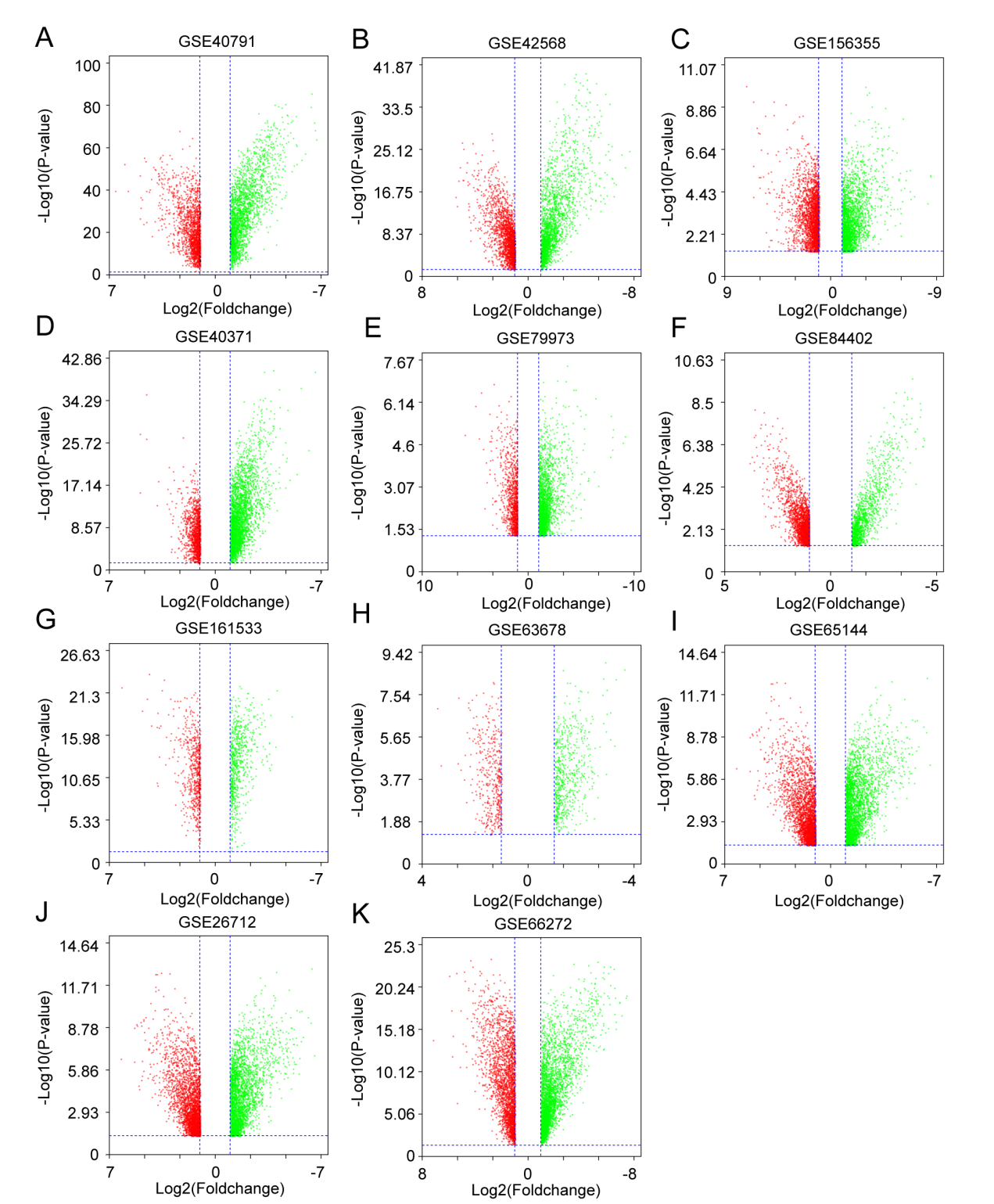


**Figure S2.** The radar map represented mRNA expression levels of GINS1 in the original 11 types of cancers from the GEO database.


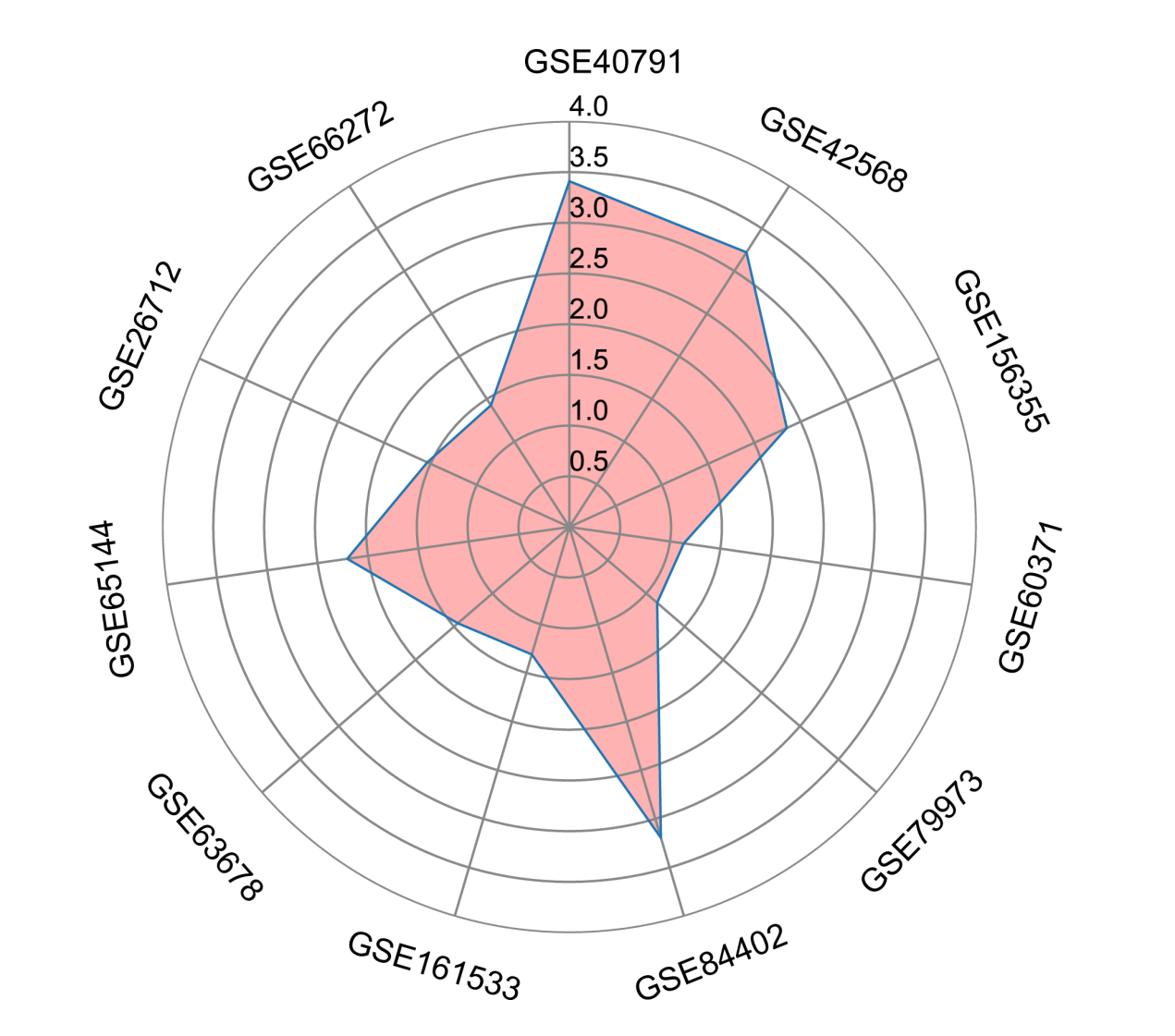


**Figure S3.**The expression analysis of GINS1 in lung cancer was conducted by the Lung Cancer Explorer Platform.


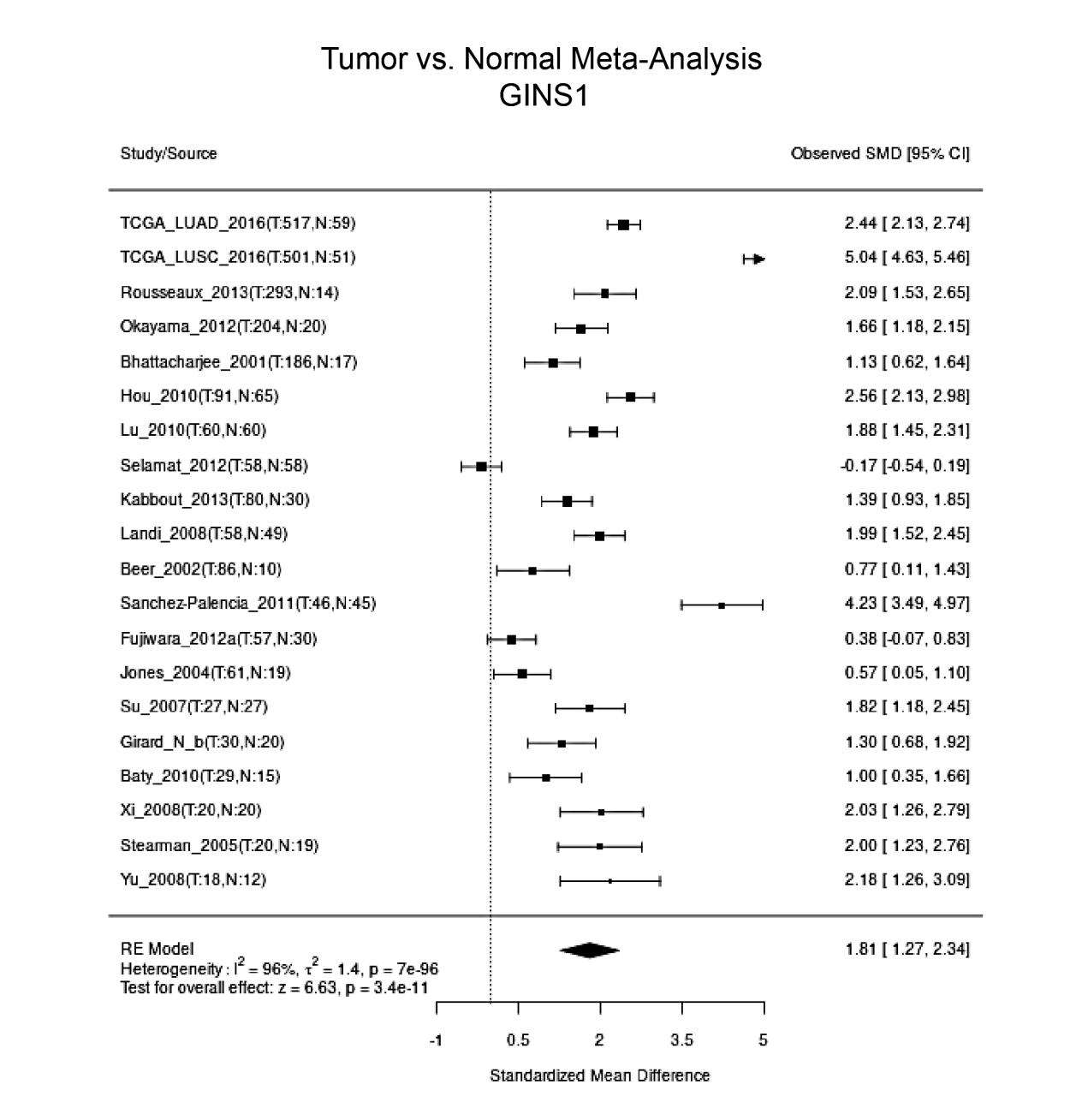


**Figure S4.** The survival analysis of GINS1 in lung cancer was conducted by the Lung Cancer Explorer Platform.


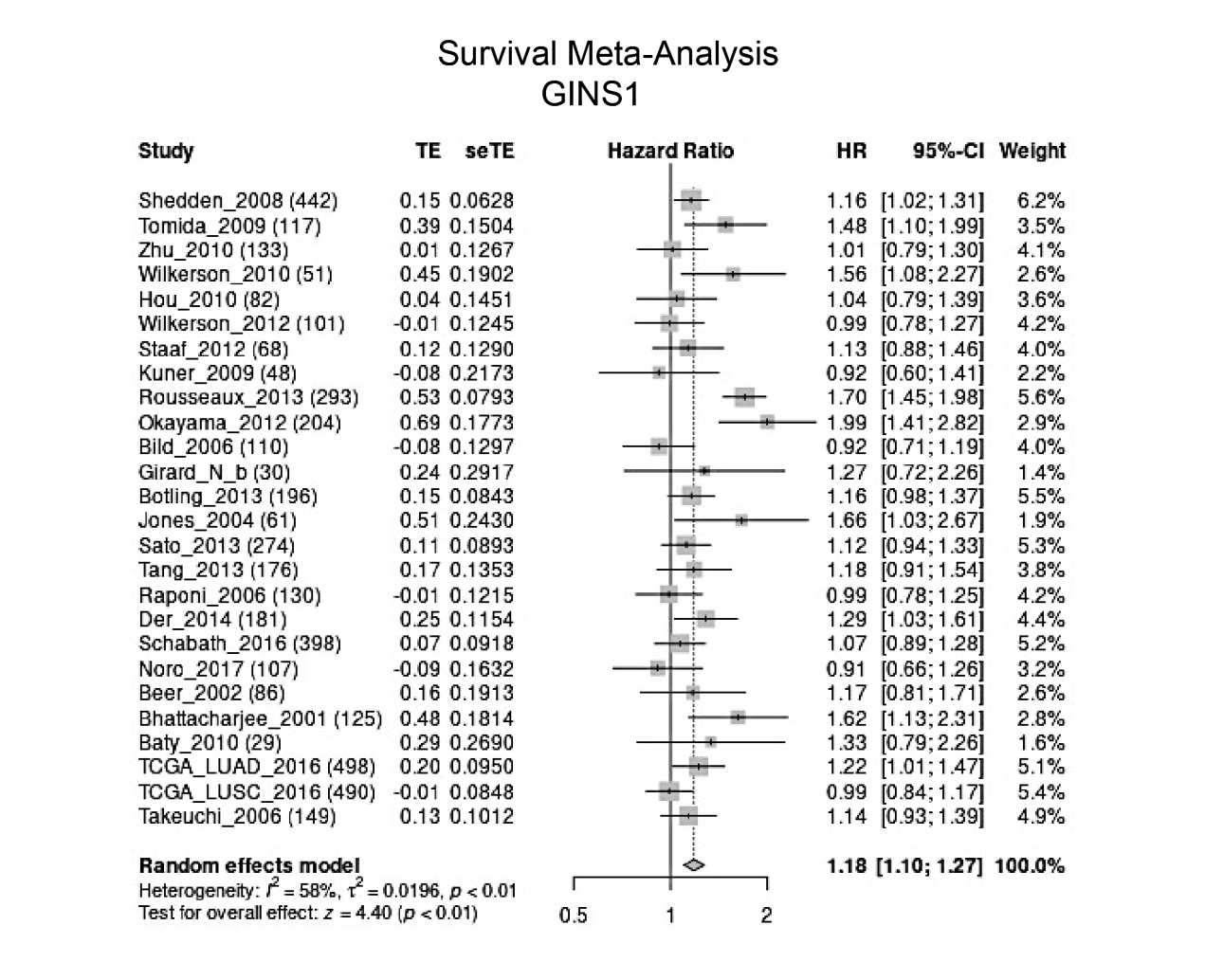


**Table SI.** The complete biological processes of these upregulated intersection genes in COVID-19 and cancers were annotated by GO analysis.

**Table SⅡ.** The Complete pathway enrichments of these upregulated intersection genes in COVID-19 and cancers were annotated by Kyoto Encyclopedia of Genes and Genomes (KEGG) analysis.

**Table SⅢ.**The potential prevention and protection compounds for cancer patients infected with COVID-19 in 11 cancer types.
